# Intelligent image analysis recognizes important orchid viral diseases

**DOI:** 10.3389/fpls.2022.1051348

**Published:** 2022-12-02

**Authors:** Cheng-Feng Tsai, Chih-Hung Huang, Fu-Hsing Wu, Chuen-Horng Lin, Chia-Hwa Lee, Shyr-Shen Yu, Yung-Kuan Chan, Fuh-Jyh Jan

**Affiliations:** ^1^ Department of Management Information Systems, National Chung Hsing University, Taichung, Taiwan; ^2^ Department of Plant Pathology, National Chung Hsing University, Taichung, Taiwan; ^3^ Advanced Plant Biotechnology Center, National Chung Hsing University, Taichung, Taiwan; ^4^ Department of Health Services Administration, China Medical University, Taichung, Taiwan; ^5^ Department of Computer Science and Information Engineering, National Taichung University of Science and Technology, Taichung, Taiwan; ^6^ Ph.D. Program in Microbial Genomics, National Chung Hsing University and Academia Sinica, Taichung, Taipei, Taiwan; ^7^ Department of Computer Science and Engineering, National Chung Hsing University, Taichung, Taiwan

**Keywords:** orchid disease, texture feature, deep learning, U-net, random forest, inception network

## Abstract

*Phalaenopsis* orchids are one of the most important exporting commodities for Taiwan. Most orchids are planted and grown in greenhouses. Early detection of orchid diseases is crucially valuable to orchid farmers during orchid cultivation. At present, orchid viral diseases are generally identified with manual observation and the judgment of the grower’s experience. The most commonly used assays for virus identification are nucleic acid amplification and serology. However, it is neither time nor cost efficient. Therefore, this study aimed to create a system for automatically identifying the common viral diseases in orchids using the orchid image. Our methods include the following steps: the image preprocessing by color space transformation and gamma correction, detection of leaves by a U-net model, removal of non-leaf fragment areas by connected component labeling, feature acquisition of leaf texture, and disease identification by the two-stage model with the integration of a random forest model and an inception network (deep learning) model. Thereby, the proposed system achieved the excellent accuracy of 0.9707 and 0.9180 for the image segmentation of orchid leaves and disease identification, respectively. Furthermore, this system outperformed the naked-eye identification for the easily misidentified categories [cymbidium mosaic virus (CymMV) and odontoglossum ringspot virus (ORSV)] with the accuracy of 0.842 using two-stage model and 0.667 by naked-eye identification. This system would benefit the orchid disease recognition for *Phalaenopsis* cultivation.

## 1 Introduction

Orchids, the flowering plants, are popular and well-received house plants worldwide. *Phalaenopsis* shared 79% of the global orchid market in 2018 (Yuan et al., 2021). *Phalaenopsis* is an important exporting flower for Taiwan. Taiwan’s *Phalaenopsis* export reached 160 million US dollars in 2021 (https://www.coa.gov.tw/). To meet the demand of the increasing share of the global orchid market, it is imperative to develop better techniques for quality control and management and the optimization of the cost for the production process. (Shaharudin et al., 2021; Yuan et al., 2021).

*Phalaenopsis* are propagated with tissue culture technique and often densely planted in greenhouses in Taiwan. Such cultivation practice can easily lead to the spread of virus diseases (Lee et al., 2021). *Phalaenopsis* are cultivated in greenhouses that provide suitable temperature, humidity, daylight, irrigation, and fertilization. However, the close planting environment in greenhouses is quite suitable for virus spread as well (Koh et al., 2014). Viruses might infect the whole orchid plants under cultivation or even spread to most orchids in the entire cultivation area. Orchids infected by viruses will greatly lose their commercial value. Hence, developing better viral disease detection protocols has always been one essential task for the quality control of orchid cultivation (Koh et al., 2014).

There are nearly 60 viruses that reportedly infect orchids. The most common viruses are odontoglossum ringspot virus (ORSV) and cymbidium mosaic virus (CymMV) worldwide (Huang et al., 2019; Lee et al., 2021). It is a common phenomenon that the coinfection of ORSV and CymMV on orchids resulted in a synergistic effect on symptoms. Capsicum chlorosis virus (CaCV), previously found on *Phalaenopsis*, was once known as “Taiwan virus”. CaCV belongs to *Orthotospovirus* which can be transmitted by thrips. These viruses frequently appear on orchid farms in Taiwan. Plants suffered from viral diseases grow slowly and eventually lose the economic values (Zheng et al., 2008; Lee et al., 2021). Early detection and removal of diseased plants is a prerequisite for ensuring the quality of orchids. The most commonly used detection methods for orchid viruses involve the nucleic acid amplification and serology. For instance, the enzyme-linked immunosorbent assay (ELISA), immunostrip test, reverse transcription-polymerase chain reaction (RT-PCR), polymerase chain reaction (PCR), and reverse transcription-loop mediated isothermal amplification (RT-LAMP) were often used (Eun et al., 2002; Chang, 2018; Lee et al., 2021). However, these detection methods are labor-intensive and expansive economically. Moreover, some of the early-stage symptoms on the orchid leaf infected by one of the common viruses are not noticeable and often not easily identified by the naked eye. Therefore, an automated and precise intelligent image analysis system for the identification of *Phalaenopsis* orchid viral diseases can be a powerful tool for better management of the viral diseases of orchids.

### 1.1 Related studies

#### 1.1.1 AI models for the identification of plant leaf diseases

During the past decade, using artificial intelligence (AI) methods with image data or big data for the prediction or detection of plant diseases has become very popular globally (Chan et al., 2018; Wu et al., 2022). For example, Ali et al. (2017) reported the detection of citrus diseases using machine learning (ML) models, k-nearest neighbor (KNN) and support vector machine (SVM), with color histogram and texture features of the infected leaf images. The classification of soybean leaf diseases was reported using the convolution neural network (CNN, a deep learning model) with the segmentation of soybean leaf and the background of each image, as well as data augmentation (by image translation and rotation) for increasing the case number of training data (Karlekar and Seal, 2020). A tomato leaf disease classification using a deep learning model, which integrated DenseNet121 and transfer learning, with conditional generative adversarial network (C-GAN) (Mirza and Osindero, 2014) for data augmentation was proposed and achieved with great accuracy (Abbas et al., 2021).

A recent review article (Dhaka et al., 2021) revealed that the AI models proposed in 20 articles for classifying different plant types (apple, cucumber, tomato, radish, wheat, rice, or maize in individual research) could reach good or excellent accuracies (0.8598-0.9970). ML-based approaches were adopted in the identification of plant growth stages, taxonomic classification of leaf images, plant image segmentation (reported in 6 studies); and deep learning (DL) architectures with transfer learning were applied in segmentation of crops and weeds, weed identification, disease classification of 12 plant species, identification of biotic and abiotic stress, and leaf counting (reported in 6 studies) (Nabwire et al., 2021).

AI models were designed to identify plant leaf diseases; they could reach fair, good, or excellent accuracies (0.590-0.9975) as mentioned in 32 articles (Dhaka et al., 2021). Basically, these researches focused on identifying leaf diseases for one of the following plant types: banana, apple, 14 crop species, 6 plant species, tomato, cucumber, rice, 25 plant species, olive, wheat, radish, 14 plant species, potato, cassava, maize, radish, grapevine, and tea (Dhaka et al., 2021). Various feature extraction methods reported in 9 studies for the image-based plant disease detection were reviewed (Ghorai et al., 2021). It was clearly documented that texture and/or color features were adopted in most of these studies on disease detection for soyabean, maize seedling, grape, bean, tomato, rice and one study on orchid (Huang, 2007; Ghorai et al., 2021).

#### 1.1.2 AI models for the orchid-associated identifications

A review article (Vishnoi et al., 2021) reported that the top three crops associated with plant disease detection using image processing techniques during 2009-2020 were rice (11%), tomato (11%), and corn (7%). Reportedly, only 1% of the studies was associated with orchids and in them there was very few studies using the AI method for the identification of common orchid diseases using orchid images (Vishnoi et al., 2021). We aimed to develop the AI system with images of orchid seedlings for accurate detection of common viral diseases of orchids. The system will provide higher accurate detection capability than that with the naked eye, and suitable for early detection that in turn can reduce the economic loss. The system enables non-destructive detection and can be developed toward automatic and fast detection that can replace the nucleic acid amplification and serology detection that are labor-intensive and neither time nor cost efficient for the comprehensive detection. Thereby, the proposed intelligence system can benefit the orchid cultivation industry.

## 2 Materials and methods

The *Phalaenopsis* images and methods described in this section were prepared and designed for accurate identification of common viral diseases of orchids. The orchid virus inoculation and *Phalaenopsis* image acquisition were described in section 2.1. The design flow of the proposed AI system, associated with the adopted image processing methods and AI models, for realizing the accurate identification of common viral diseases of orchids using the acquired *Phalaenopsis* images was sequentially described in section 2.2.

### 2.1 Image dataset

#### 2.1.1 Orchid virus inoculation

Leaves of ORSV- or CaCV-infected *N. benthamiana* and CymMV-infected *C. quinoa* were ground in phosphate buffer (0.1 M potassium phosphate buffer, pH 7.0) and the resultant saps were inoculated to *Phaleanopsis* seedlings. Inoculated plants were maintained in a growth chamber with the temperature set at 25°C located in the National Chung Hsing University (NCHU, Taichung, Taiwan) for symptom development.

#### 2.1.2 Samples and photographic images

**Figure 1:**
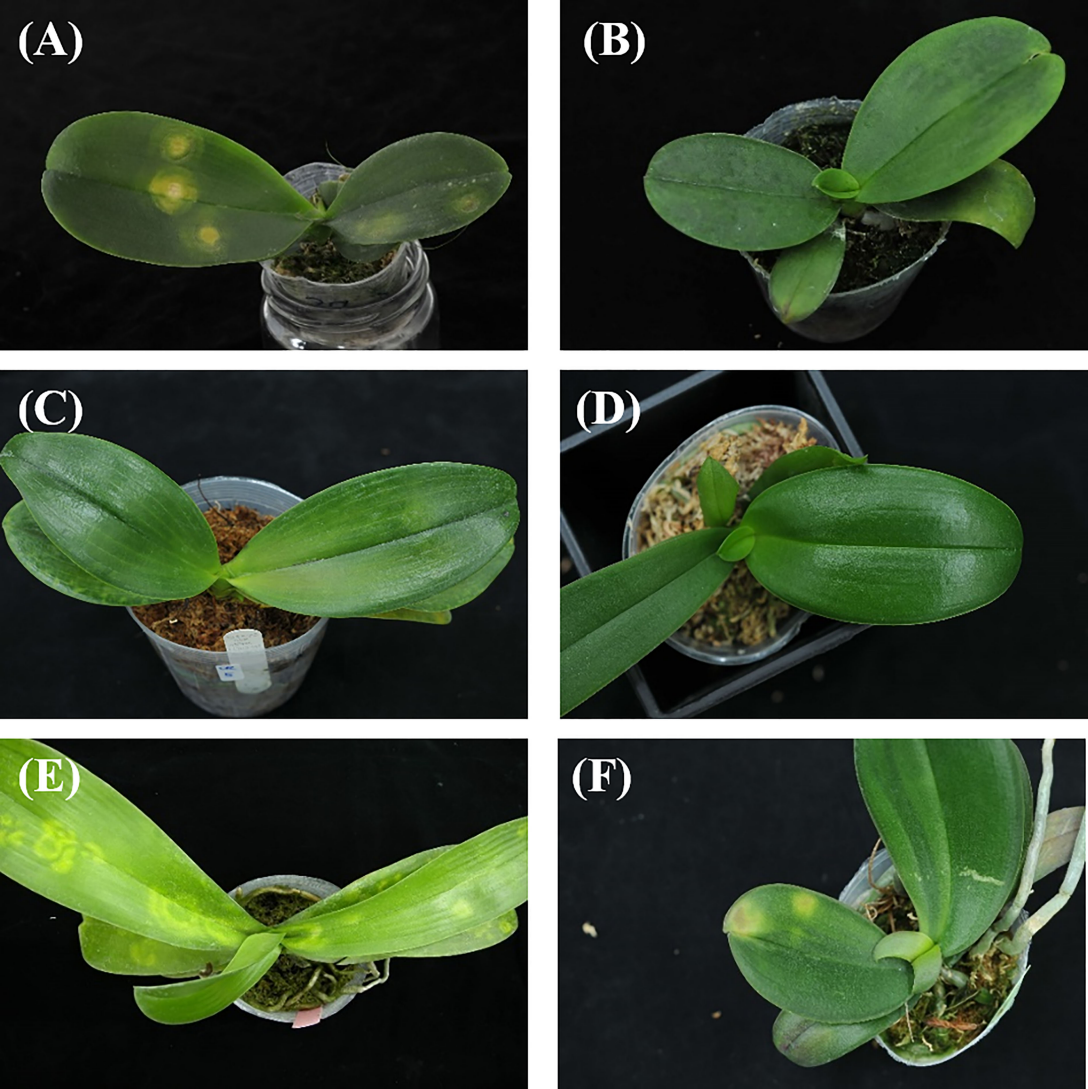
Image examples of orchids: **(A)** Capsicum chlorosis virus (CaCV) infection, **(B)** Cymbidium mosaic virus (CymMV) infection, **(C)** Odontoglossum ringspot virus (ORSV) infection, **(D)** health, **(E)** and **(F)** others.

A total of 1888 images of *Phalaenopsis* consisted of five categories (CymMV infection, ORSV infection, CaCV infection, health and others) were generated. Photographic samples included collections from various orchid farms and artificially inoculated *Phalaenopsis*. The virus-infected *Phalaenopsis* was photographed with a digital single-lens reflex (DSLR) camera (Nikon D300). Plants were placed on black cloth for photos from different angles. **Figure 1** shows examples of these images. The study viruses were inoculated into *Phalaenopsis* mechanically. ORSV-infected plants exhibited chlorotic mosaic symptoms on the apical leaves. CymMV-infected plants exhibited mosaic symptoms on the apical leaves. CaCV-infected plants exhibited chlorotic ringspots symptoms on the inoculated leaves. All samples were detected by ELISA for the presence of viruses. ORSV infection induced symptoms of chlorotic mosaic, mosaic, chlorotic arch and chlorotic spots on *Phalaenopsis* leaves. CymMV infection induced symptoms of chlorotic necrosis, mosaic, necrotic streaks, and necrotic spot turned dark on *Phalaenopsis* leaves. CaCV infection induced symptoms of chlorotic ring spots of different sizes on *Phalaenopsis* leaves. In Taiwan, eight viruses reportedly infect orchids. The most common orchid viral diseases in Taiwan are ORSV, CymMV, and CaCV. *Phalaenopsis* orchids showing virus-like symptoms were collected in Taiwan. All samples were detected by ELISA for the presence of viruses. *Phalaenopsis* leaves with virus-like symptoms but from which no virus was detected were listed in the category of “others”. In other words, symptomatic leaves other than CymMV-, ORSV-, and CaCV-infected leaves were listed in the category as “others”.

### 2.2 System architecture and design methods

**Figure 2:**
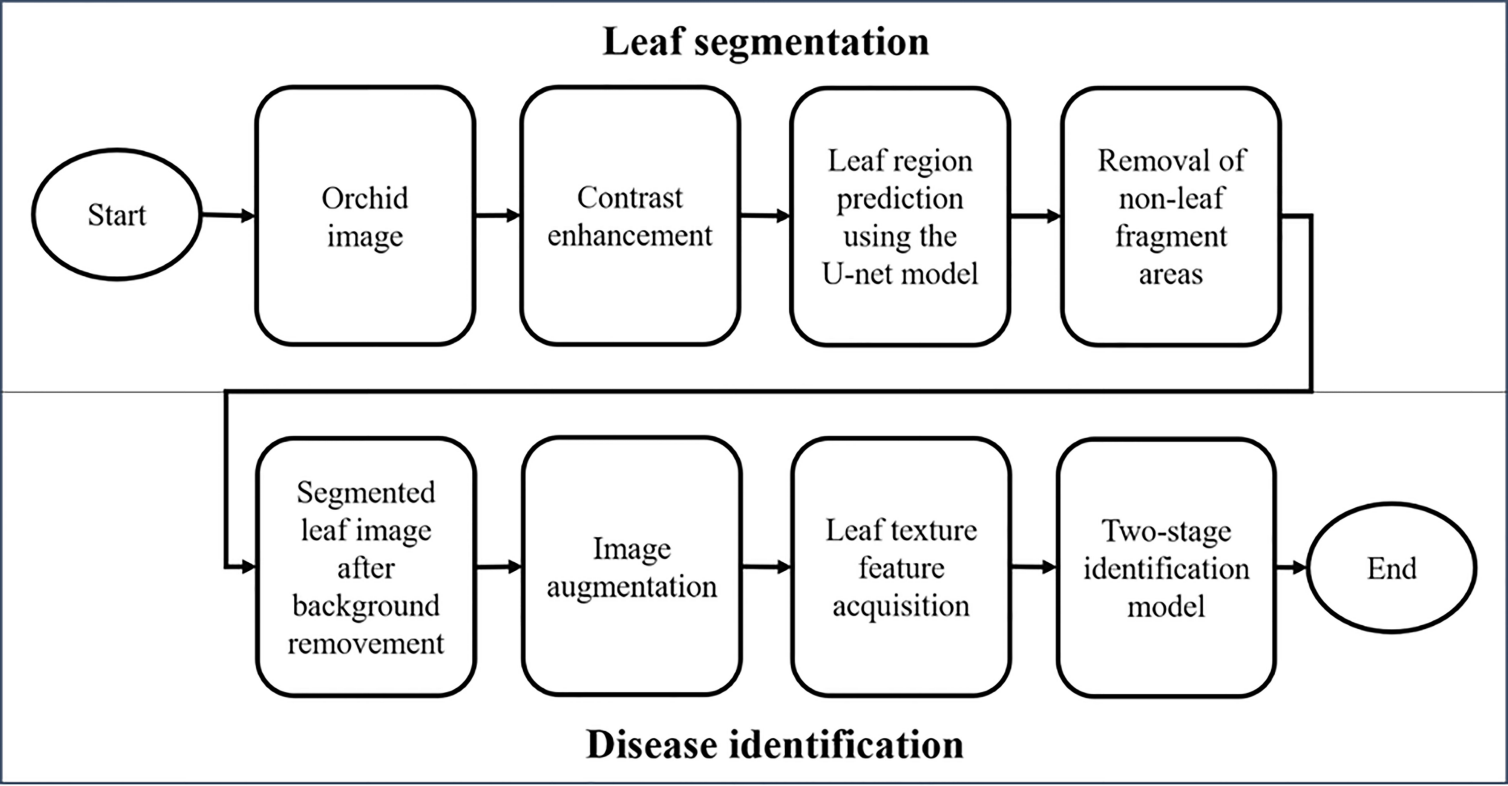
The proposed system architecture for identifying orchid diseases.

The system architecture for the identification of *Phalaenopsis* orchid diseases (shown in **Figure 2**) included two major portions, namely, the leaf segmentation and disease identification. The leaf segmentation can keep only the leaf image with all other parts being removed from a *Phalaenopsis* orchid image. The leaf segmentation can be regarded as the image preprocessing before the disease identification. The disease identification, following the leaf segmentation, was designed to identify the five categories (described in section 2.1) using the two-stage-AI model with the segmented leaf image and the acquired leaf texture features of the *Phalaenopsis* orchid image. The proposed AI system performed all the procedures sequentially, shown in the 8 blocks in **Figure 2**, for the identification of orchid diseases. The design methods of this identification system, illustrated in **Figure 2**, were described in detail in the following subsections. The detailed procedures and methods of two major portions, the leaf segmentation and disease identification, were described in section 2.2.1 and section 2.2.2, respectively, with each subsection title corresponding to the block in **Figure 2**. For example, the subsection title “Contrast enhancement” follows the first paragraph of section 2.2.1 is also presented as one procedure shown in the second left block in **Figure 2**. The details of each procedure are described after the corresponding subsection title. This identification system, including AI models and image processing approaches, were developed using Python 3.6.10 (with Tensorflow 1.15 and Keras) under Windows 10 pro and executed in Intel i7-9700K 8-core CPU and Nvidia RTX-2080TI GPU.

#### 2.2.1 Leaf segmentation

The first half of this system architecture, illustrated in the upper half of **Figure 2**, mainly performs the leaf segmentation from each *Phalaenopsis* orchid image by the contrast enhancement (CE) using color space transformations and gamma correction for solving the non-uniform brightness issue of the input images; leaf region prediction using the U-net model for selecting the leaf regions in an orchid image; and removal of non-leaf fragment areas using connected component labeling for removing small fragment areas and keeping the desired leaf regions in an orchid image. Technical details of these procedures are explained as follows.

### Contrast enhancement

The non-uniform brightness of input images was a common issue in the field of image processing. The following image preprocessing method, the contrast enhancement with color space transformations was adopted for brightness adjustment in this study. A color image is usually expressed using the RGB format with each pixel range from 0-255 levels for red, green, and blue components, respectively. The RGB format can be transferred into the YUV space using Equation (1) (wikipedia.org, 2022). In the YUV space, Y, U, and V components represent the luminance (or brightness), chrominance, and chroma, respectively. The YUV space can also be transferred back to the RGB space by Equation (2) (wikipedia.org, 2022).


(1) $$\left[\begin{array}{c} \text{Y}\\ \text{U}\\ \text{V} \end{array}\right]=\left[\begin{array}{ccc} 0.299 & 0.587 & 0.114\\ -0.169 & -0.331 & 0.5\\ 0.5 & -0.419 & -0.081 \end{array}\right]\left[\begin{array}{c} \text{R}\\ \text{G}\\ \text{B} \end{array}\right]+\left[\begin{array}{c} 0\\ 128\\ 128 \end{array}\right]$$

(2) $$\left[\begin{array}{c} \text{R}\\ \text{G}\\ \text{B} \end{array}\right]=\left[\begin{array}{ccc} 1 & -0.00093 & 1.401687\\ 1 & -0.3437 & -0.71417\\ 1 & 1.72216 & 0.00099 \end{array}\right]\left[\begin{array}{c} \text{Y}\\ \text{U}-128\\ \text{V}-128 \end{array}\right]$$

The gamma correction, expressed in Equation (3), can be used to adjust brightness level within an image by computing a power of gamma *γ* of the normalized brightness from Y-component *I*_*Y*_(*x*,*y*) , brightness value for each pixel within the image. If *γ* is smaller than 1, the detail of the dark region can be enhanced (highlighted). If *γ* is larger than 1, the detail of the bright region can be enhanced.


(3) $$I_{Y'}\;={\left(\frac{I_{Y}\left(x,y\right)-min\;}{max-min}\right)}^{\gamma }\times 255$$

**Figure 3:**
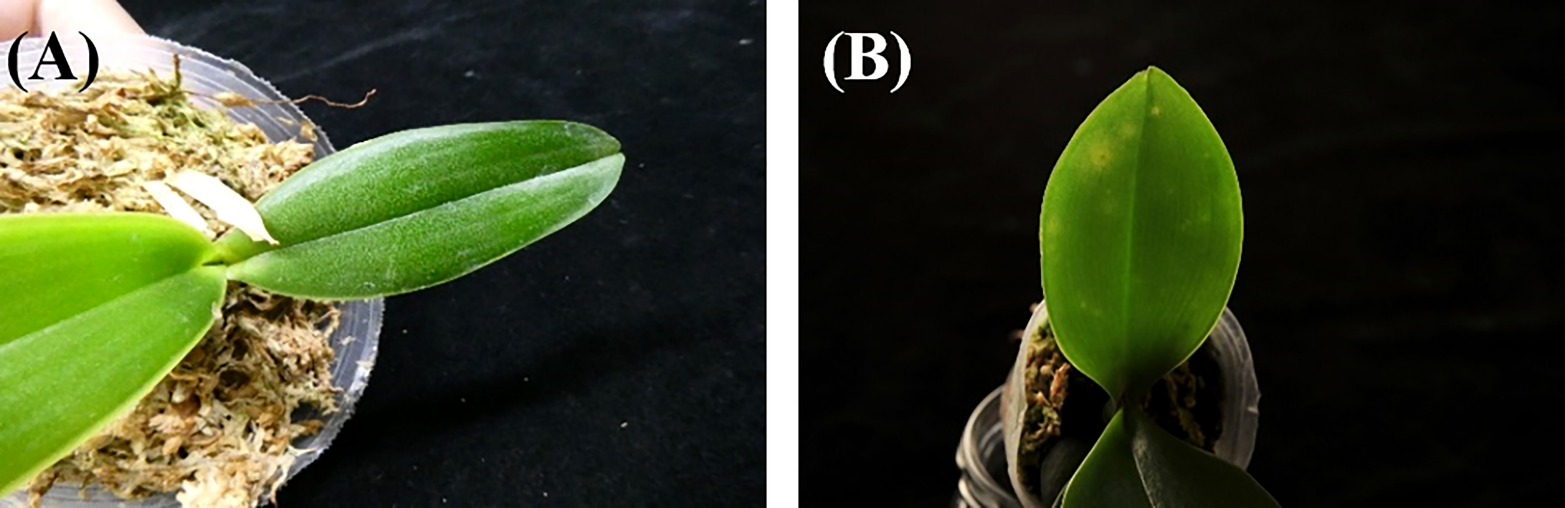
The bright **(A)** and dark **(B)** orchid images.

**Figure 4:**
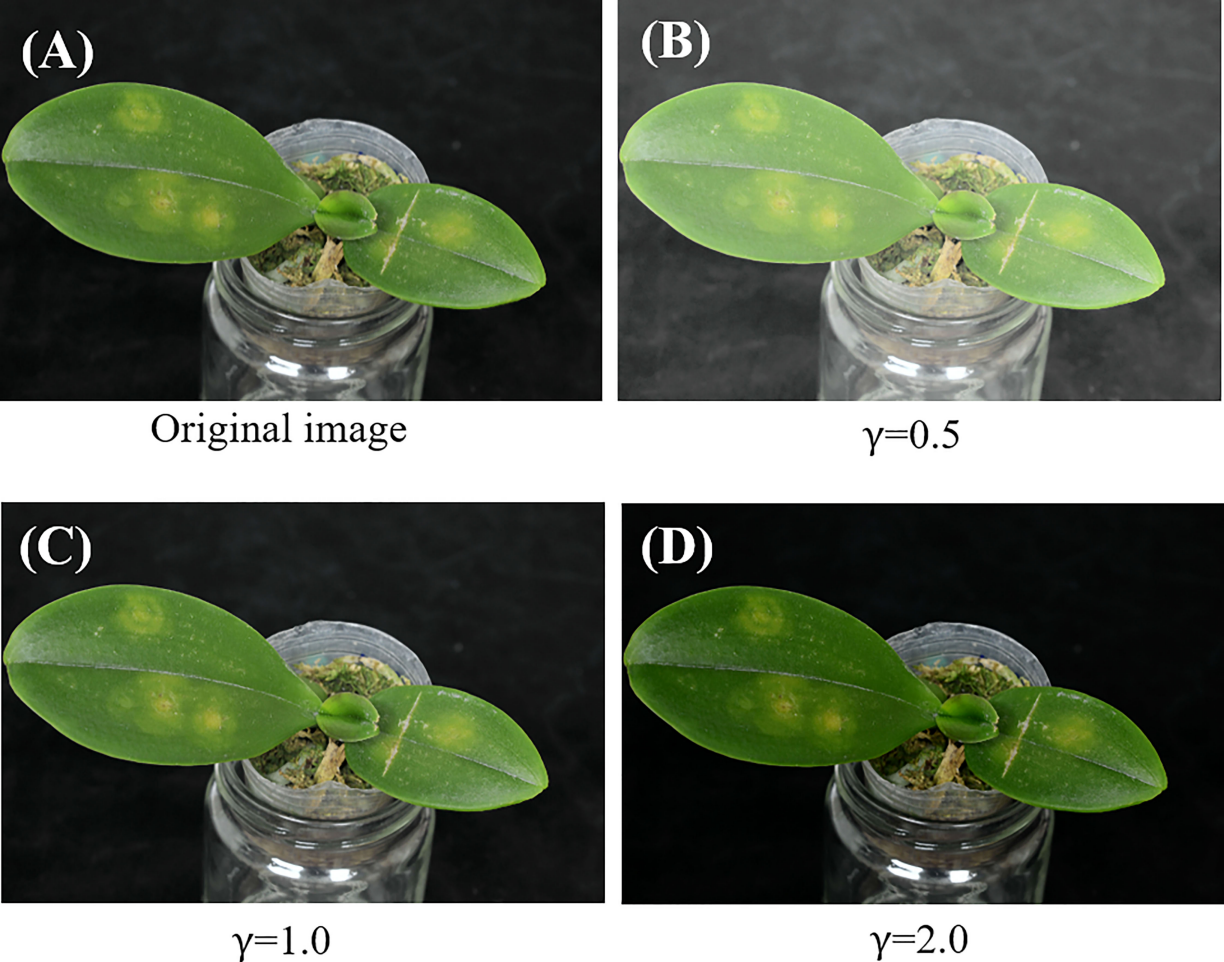
**(A)** An original orchid image and **(B-D)** images after contrast enhancement with different gamma values.

The brightness of the input *Phalaenopsis* orchid images may not be uniform. Some of the *Phalaenopsis* orchid images may be bright and some may be dark as shown in (**Figures 3A, B**) respectively. Because of this issue, a color *Phalaenopsis* orchid image was read in and followed by the contrast enhancement. The contrast enhancement was performed by transferring the RGB space of the input image, such as shown in (**Figure 4A**), into the YUV space using Equation (1) and followed by the gamma correction, using Equation (3), for obtaining the enhanced Y component *I*_*Y*'_. The U and V components (chrominance and chroma) were remained the same to keep the color information of each orchid image including symptom colors and symptom textures of each leaf image. **Figures 4B–D** shows the images after the contrast enhancement with different gamma values.

### Leaf region prediction using the U-net model

In order to prevent objects unrelated to leaf symptoms such as the soil and potted container from affecting the subsequent model of disease identification, we separated the *Phalaenopsis* orchid leaf region from the image using a U-net model before performing the disease identification.

The U-net (Ronneberger et al., 2015) was a very popular AI network architecture for image segmentation. Because the network architecture of the model is very similar to a capital letter U, hence named U-net. U-net consists of two portions, namely the encoder and decoder. The encoder mainly extracts image features of different sizes by multiple sub-models. The decoder concatenates features from the same level layers of the encoder and up sample to generate the trained image mask. The U-net features performing the image segmentation with a small amount of training images.

**Figure 5:**
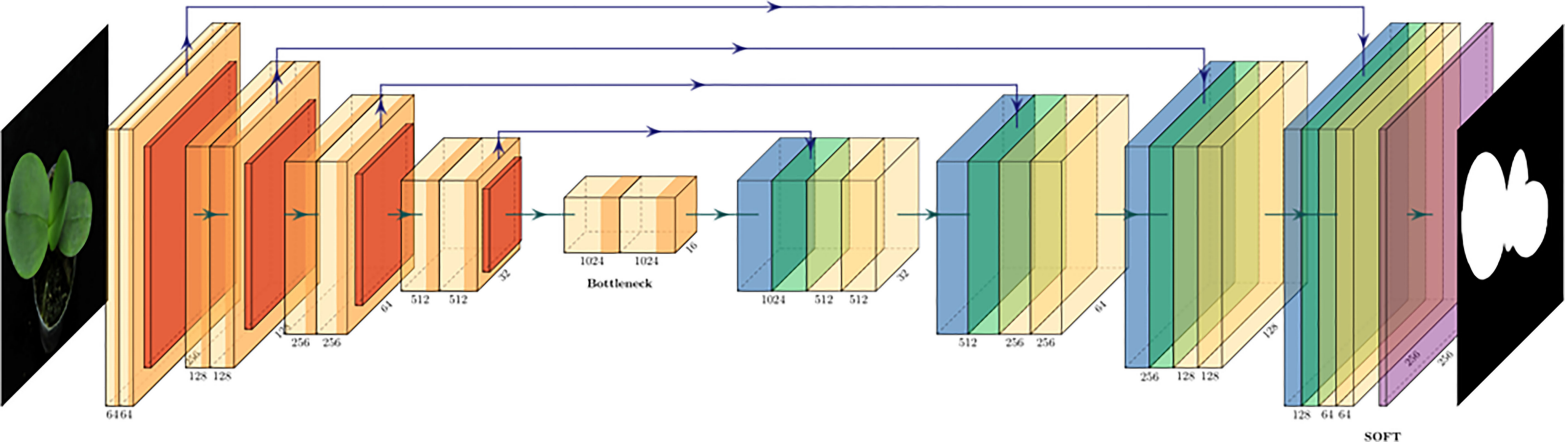
The U-net model for automatic leaf region prediction.

**Figure 6:**
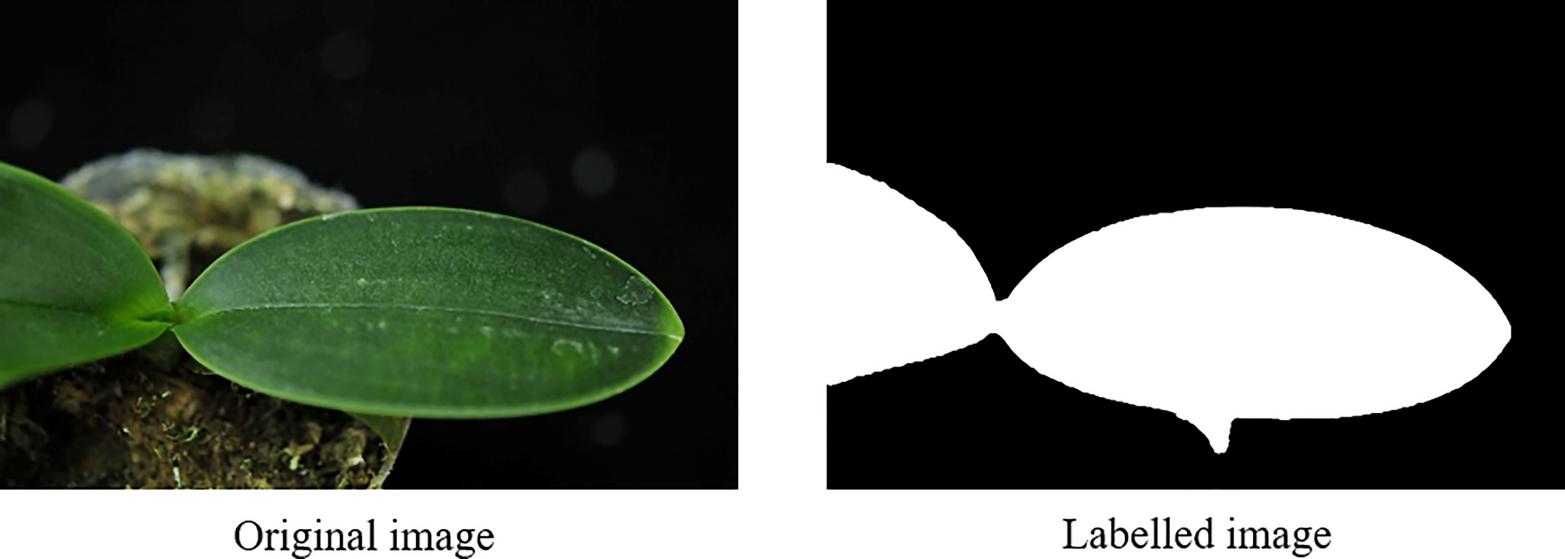
Manual labelling for the leaf region (white region) and background region (black region).

The U-net model adopted in this study was illustrated in **Figure 5**. The leaf region and background region were specified in white and black colors, respectively, (as illustrated in **Figure 6**) using an image labeling tool for preparing the training data. Then the output of the trained U-net model will provide the leaf region and background region. 1504 and 377 images (randomly split from 1881 *Phalaenopsis* orchid images) were used as the training dataset (80%) and testing dataset (20%), respectively, for designing the U-net model. U-net parameters adopted in this study include that image size=256x256, 3 channels, batch size=8, epoch=200, Adam optimizer, and learning rate=0.0001.

The contrast enhanced YUV space after gamma correction, using Equation (3), with different values of *γ* were transferred back to the RGB space using Equation (2) and input to the U-net model for obtaining the best value of *γ* .

The performances of segmenting the leaf region from the background region in a *Phalaenopsis* orchid image using the U-net model were quantified by the parameters formulated in Equations (4)-(7). TP, TN, FP and FN represent for the true positive, true negative, false positive, and false negative, respectively.


(4)  Accuracy = ( TP + TN ) / Total Number

(5) Precision = TP / ( TP + FP )

(6) Recall = TP / ( TP + FN ) 

(7) F1 Score=2×Precision×Recall/(Precision+Recall)

### Removal of non-leaf fragment areas

**Figure 7:**
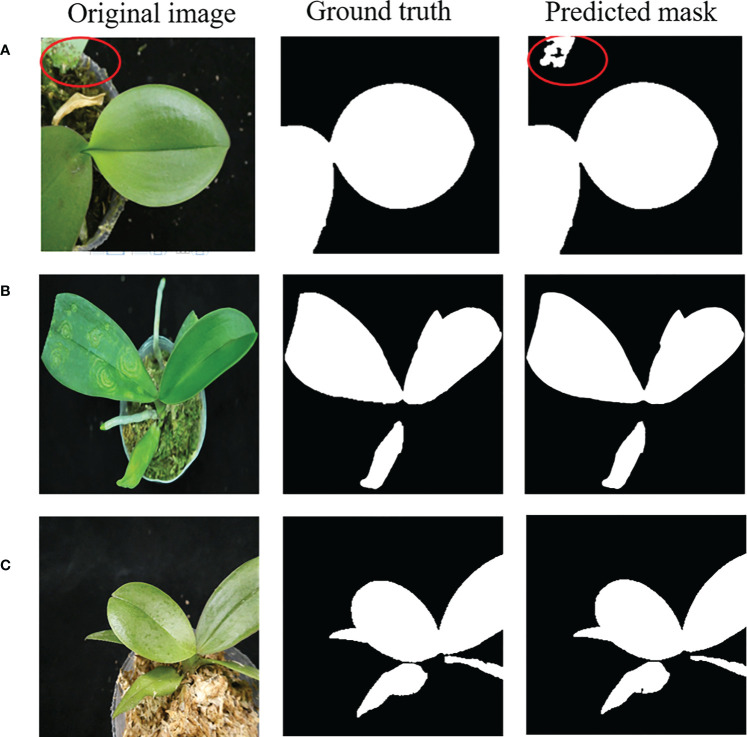
**(A–C)** Examples of the predicted leaf region (mask).

After the contrast enhancement and leaf region prediction using the U-net model, the mask region generated may include non-leaf regions such as the white mask region indicated by the red oval in the right panel of (**Figure 7A**). Besides that, the leaf regions in an image may not be connected as shown in (**Figure 7**B). Furthermore, sometimes the side-view picture of a leaf was taken, such as that shown in the left panel of (**Figure 7**C), and its top-view leaf texture may not be observed. Therefore, the predicted mask of a side-view leaf may be less helpful for the disease identification.

**Figure 8:**
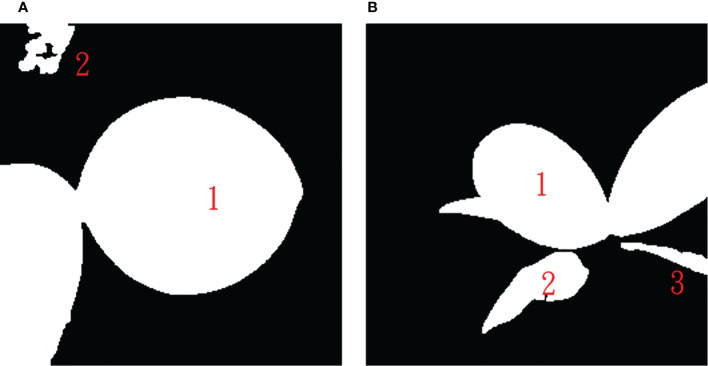
Results after performing the connected component labeling with **(A)** two connected regions and **(B)** three connected regions.

U-net predicted leaf regions (mask), such as shown in the right three panels in **Figure 7**, were further processed using the connected component labeling method with 8-neighbor connectivity (Chen et al., 2018) to recognize each connected region to obtain multiple connected regions in an *Phalaenopsis* orchid image. **Figure 8** shows two examples of the automatic labeled connected regions with consecutive Arabic numerals after performing the connected component labeling.

The maximum area, *max*_area_, of connected leaf regions obtained was computed (excluding the background region). If the area of any of the rest connected regions is smaller than a threshold area *TH*_area_ (a ratio of *max*_area_), this connected region was regarded as a non-leaf fragment area and will be removed from the predicted leaf region. Experimental results obtained in this study suggested that *TH*_area_=1/20 *max*_area_ could have the highest accuracy for the automatic removal of non-leaf fragment regions and leaf segmentation.

#### 2.2.2 Disease identification

The second half of this system architecture, illustrated in the lower half of **Figure 2**, mainly performs the disease identification from segmented leaf images by sequentially performing the image augmentation for obtaining more and balanced numbers of images among all categories, leaf texture feature acquisition for obtaining and quantifying symptom features on each leaf image, and two-stage identification model for accurate disease identification. Technical details of the procedures were described as follows.

### Image augmentation

**Figure 9:**
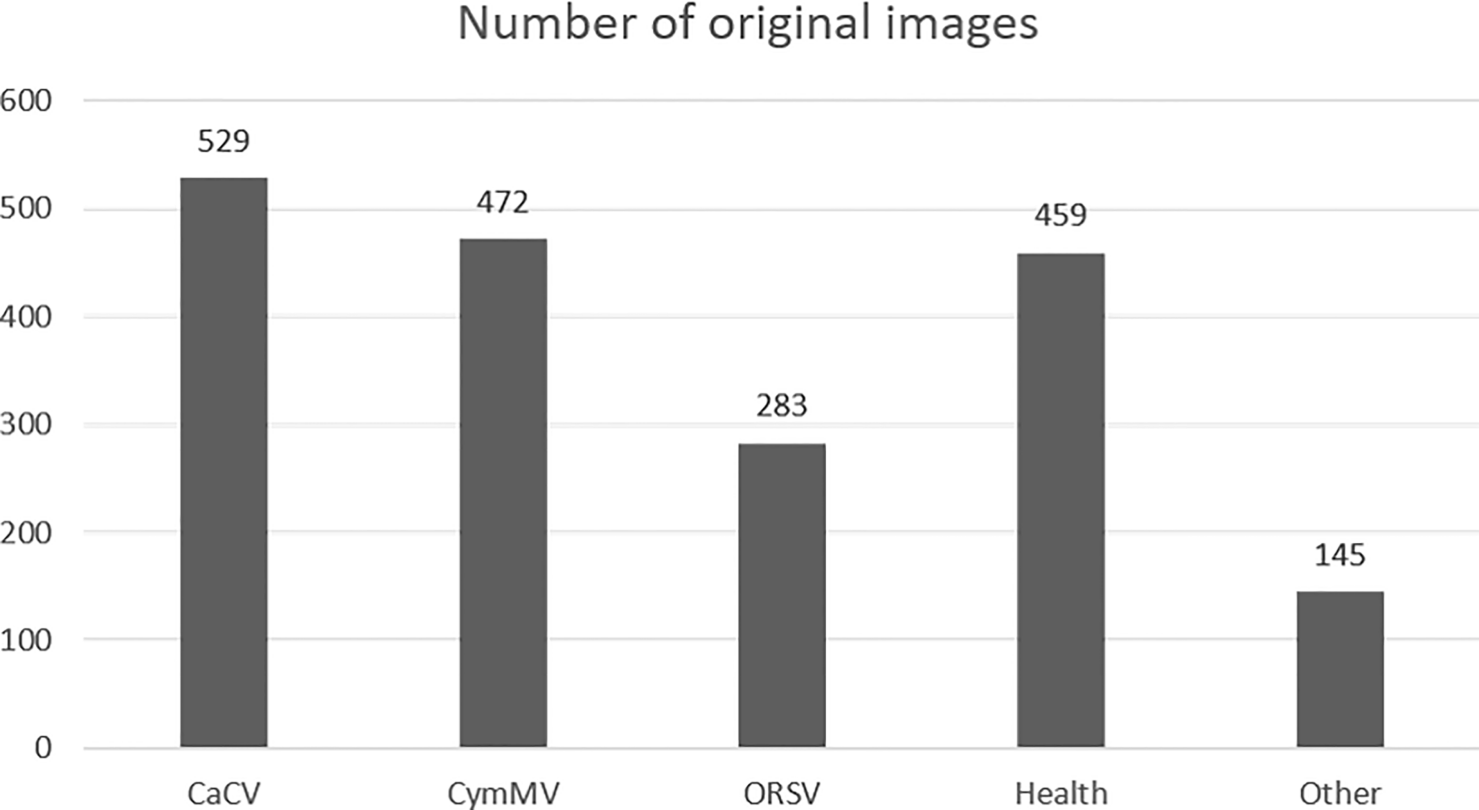
The number of collected orchid images with different diseases.

The numbers of five categories of *Phalaenopsis* orchid images collected in this study were imbalanced as shown in **Figure 9**. The data augmentation was performed to solve the imbalanced issue and avoid overfitting for designing a better identification model. The smaller number categories, ORSV and “others”, were augmented by randomly selecting some images and rotating them by ±15° . The image numbers of ORSV and “others” were increased by 0.5 and 2 folds, respectively. More than 400 images for each category were obtained.

### Leaf texture feature acquisition

The texture features of the *Phalaenopsis* orchid leaf were computed and extracted using two techniques: the method of rotation invariant local binary pattern (LBP^ri^) (Ojala et al., 2002) and the method of gray level co-occurrence matrix (GLCM) (Haralick et al., 1973).

The technique of LBP^ri^ can provide the rotation invariant features using a local binary pattern, LBP, (Ojala et al., 2002). The computation of LBP^ri^ is shown in Equation (8). LBP^ri^ creates  P -bit circular patterns and encoded them as P -bit binary numbers for a point with P circular neighbors (with radius R ). The minimum value of all rotation results is selected as the LBP value of the pattern center. For R =1 and P =8, 36 rotation invariant patterns (features) can be obtained and were used as 36 features for leaf texture in the current study.


(8) $${\text{LBP}}_{\text{P},\text{R}}^{\text{ri}}=\min\left\{\text{ROR}\left({\text{LBP}}_{\text{P},\text{R}},\text{k}\right)\text{∣k}=0,\ldots ,\text{P}-1\right\}$$

where ROR(LBP_P,R_,k) rotates k bit clockwise with radius R and P neighbors of a circular pattern LBP (Ojala et al., 2002).

The method of GLCM can extract and quantify multiple features for the texture of a gray level image (Haralick et al., 1973; Ozdemir et al., 2008; Zulpe and Pawar, 2012; Hall-Beyer, 2017; Andono et al., 2021). Six features, contrast, dissimilarity, homogeneity, angular second moment (ASM), energy, and correlation, were computed using GLCM with Equations (9)-(14) (Haralick et al., 1973; Ozdemir et al., 2008; Zulpe and Pawar, 2012; Hall-Beyer, 2017; Andono et al., 2021) in the current study. In the matrix of GLCM, the matrix element, *P*_*d*,*θ*_(*i*,*j*), represents the relative occurrence frequency for the gray level pair with gray level *i* and *j* among all pixel pairs separated with distance *d* and relative angle *θ* in a gray level image.


(9) $$\text{Contrast }=\sum_{n=0}^{L_{g}-1}n^{2}\left\{\sum_{i=0}^{L_{g}-1}\sum_{j=0}^{L_{g}-1}P_{d,\theta }\left(i,j\right)\right\},\left|i-j\right|=n$$

(10) $$\text{Dissimilarity }=\sum_{i=0}^{L_{g}-1}\sum_{j=0}^{L_{g}-1}P_{d,\theta }\left(i,j\right)\left|i-j\right|$$

(11) $$\text{Homogeneity }=\sum_{i=0}^{L_{g}-1}\sum_{j=0}^{L_{g}-1}\frac{P_{d,\theta }\left(i,j\right)}{+{\left(i-j\right)}^{2}}$$

(12) $$\text{ASM }=\sum_{i=0}^{L_{g}-1}\sum_{j=0}^{L_{g}-1}P_{d,\theta }{\left(i,j\right)}^{2}$$

(13) $$\text{Energy }=\sqrt{\text{ASM}}$$

(14) $$\text{Correlation }=\;\sum_{i=0}^{L_{g}-1}\sum_{j=0}^{L_{g}-1}\left(\frac{\left(i\times j\right)P_{d,\theta }\left(i,j\right)-\mu_{x}\mu_{y}}{\sigma_{x}\sigma_{y}}\right)$$

where *μ*_*x*_ , *μ*_*y*_ *σ*_*x*_ , and *σ*_*y*_ are the means and standard deviations of $P_{x}\left(i\right)=\sum_{j=0}^{L_{g}-1}P_{d,\theta }\left(i,j\right)$ , and $P_{y}\left(j\right)=\sum_{i=0}^{L_{g}-1}P_{d,\theta }\left(i,j\right)$ , respectively.

Each of the six features was computed for four different angles (*θ*=0^°^,45^°^,90^°^,and 135^°^ ) with distance *d*=1 , as mentioned in the method of GLCM (Haralick et al., 1973) and select the maximum value as this feature as shown in Equation (15).


(15) $$\text{Feature}=\text{max}\left(\text{feature }0^{{}^{\circ}},\text{feature }45^{{}^{\circ}},\text{feature }90^{{}^{\circ}},\text{feature }135^{{}^{\circ}}\right)$$

Totally 42 features of the leaf texture were extracted from a *Phalaenopsis* orchid image using methods of LBP^ri^ and GLCM, and each feature was normalized within 0~1.

### Two-stage identification model

**Figure 10:**
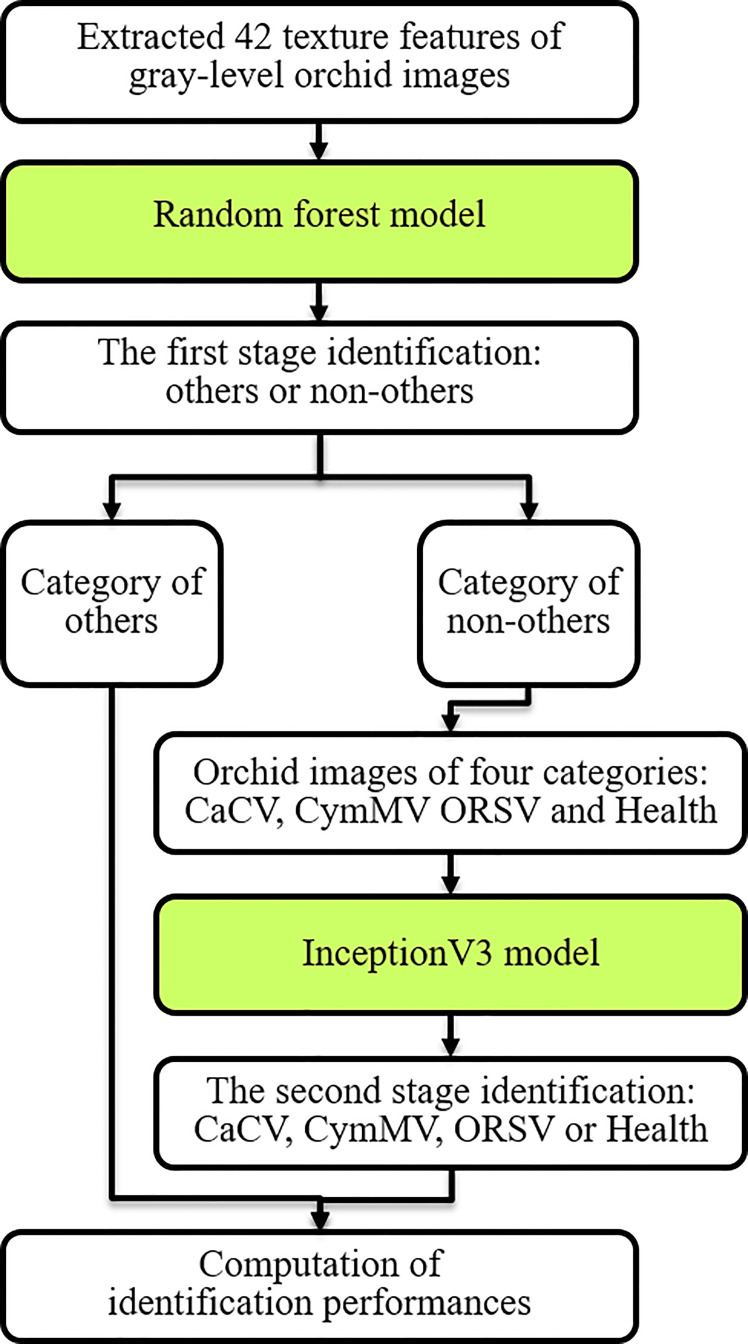
Procedures of the two-stage identification model for identifying orchid diseases using extracted leaf features and orchid images.

**Figure 11:**
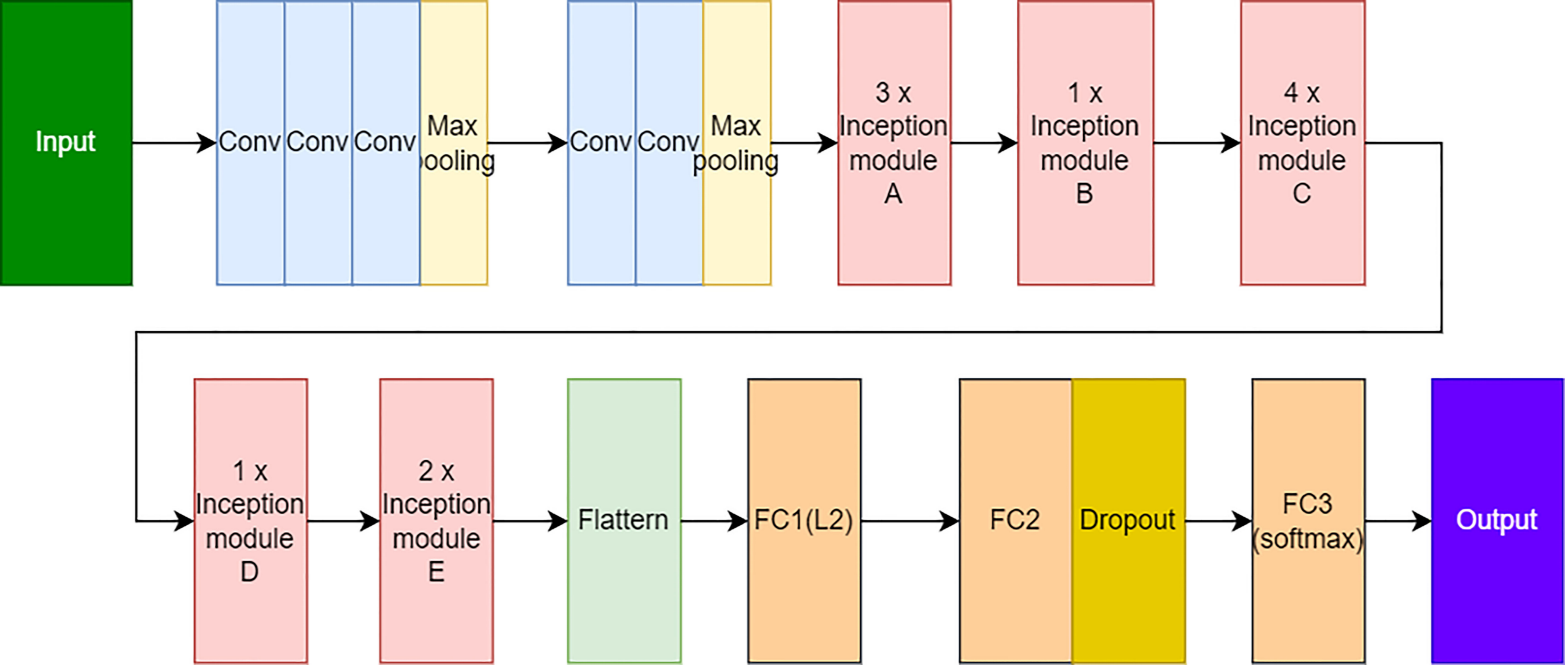
The network structure of designed InceptionV3 model.

We adopted and proposed the two-stage model as shown in **Figure 10** for *Phalaenopsis* orchid disease identification with excellent identification performances. Firstly, a random forest (RF) model (Breiman, 2001) was designed using 42 features of leaf texture obtained using methods of LBP^ri^ and GLCM to preliminarily classify the 5 categories (mentioned in section 2.1) into 2 categories: “others” and “non-others”. Secondly, the InceptionV3 model (Bal et al., 2021), a deep learning model as illustrated in **Figure 11**, was created using the *Phalaenopsis* orchid images to identify an input image is the CaCV-infected, CymMV-infected, ORSV-infected or healthy leaf.

The random forest model (Breiman, 2001; Athey et al., 2019; Musolf et al., 2021; Singh et al., 2021; Yang et al., 2021; Al-Mamun et al., 2022) is one of a popular machine learning models for classification. Before the first stage of the identification model, the R, G and B components of each *Phalaenopsis* orchid image were individually transferred to a gray level image and the methods of LBP^ri^ and GLCM described above were used to obtain the texture features of each gray-level image. There were 1841 (80%) and 462 (20%) images for training and testing, respectively. Three datasets of texture features corresponding to the transferred gray-level image of R, G, or B component of all these images were extracted respectively. Each of the three datasets was used to design and verify the identification performance of a random forest model with 300 decision trees individually.

Recently, the feature importance of a RF model was increasingly presented in AI applications (Musolf et al., 2021; Algehyne et al., 2022; Loecher, 2022). The package of gini importance (or mean decrease impurity) built in Python (scikit-learn package) was adopted for the feature importance computation of the RF model (Menze et al., 2009; Wei et al., 2018; Płoński, 2020). Furthermore, the method of principal component analysis, PCA, (Bro and Smilde, 2014; Zhao et al., 2019) was adopted to reduce the dimension of data features to analyze and visualize the distribution of the most important features among all categories for category selection in the first stage design.

An InceptionV3 model, the third generation of GoogleNet Inception, can excellently extract the detail information and feature of an image, reduce training parameters, and solve overfitting issue by the modified neural structures (Gunarathna and Rathmayaa, 2020; Jayswal and Chaudhari, 2020; Bal et al., 2021; Xiao et al., 2022). In the second stage of the disease identification model, an InceptionV3 model was designed as the identification structure shown in **Figure 11**. The feature extraction portion of the inceptionV3 model included the convolution layers, pooling layers, and inception model layers A-E as illustrated in **Figure 11**, and was followed by classification layers (which sequentially included two fully connected layers (FC1 with L2 normalization and FC2), a dropout layer (for solving the overfitting issue during training), and the third fully connected layer (FC3) with the softmax activation function) for classification (Bal et al., 2021). The flatten layer (Bal et al., 2021) was adopted for connecting the feature extraction portion to the classification layers.

An inceptionV3 model pretrained using the ImageNet dataset (which includes 1000 categories for object identification) was selected and transferred to the inceptionV3 model, illustrated in **Figure 11**, for initializing hyper parameters (Russakovsky et al., 2015; Chen et al., 2019; Gunarathna and Rathmayaa, 2020; Morid et al., 2021). In this study, 1841 (80%) and 462 (20%) images were adopted for further training and testing the InceptionV3 model. The output category of inceptionV3 model for each image was determined by the category with the highest confidence (among the four categories). Adopted parameters of the optimized InceptionV3 model included the input image size=224x224, 3 channels, batch size=16, epoch=500, Adam optimizer, and learning rate=0.000005.

## 3 Experimental results

### 3.1 Performances of the leaf segmentation

**Figure 12:**
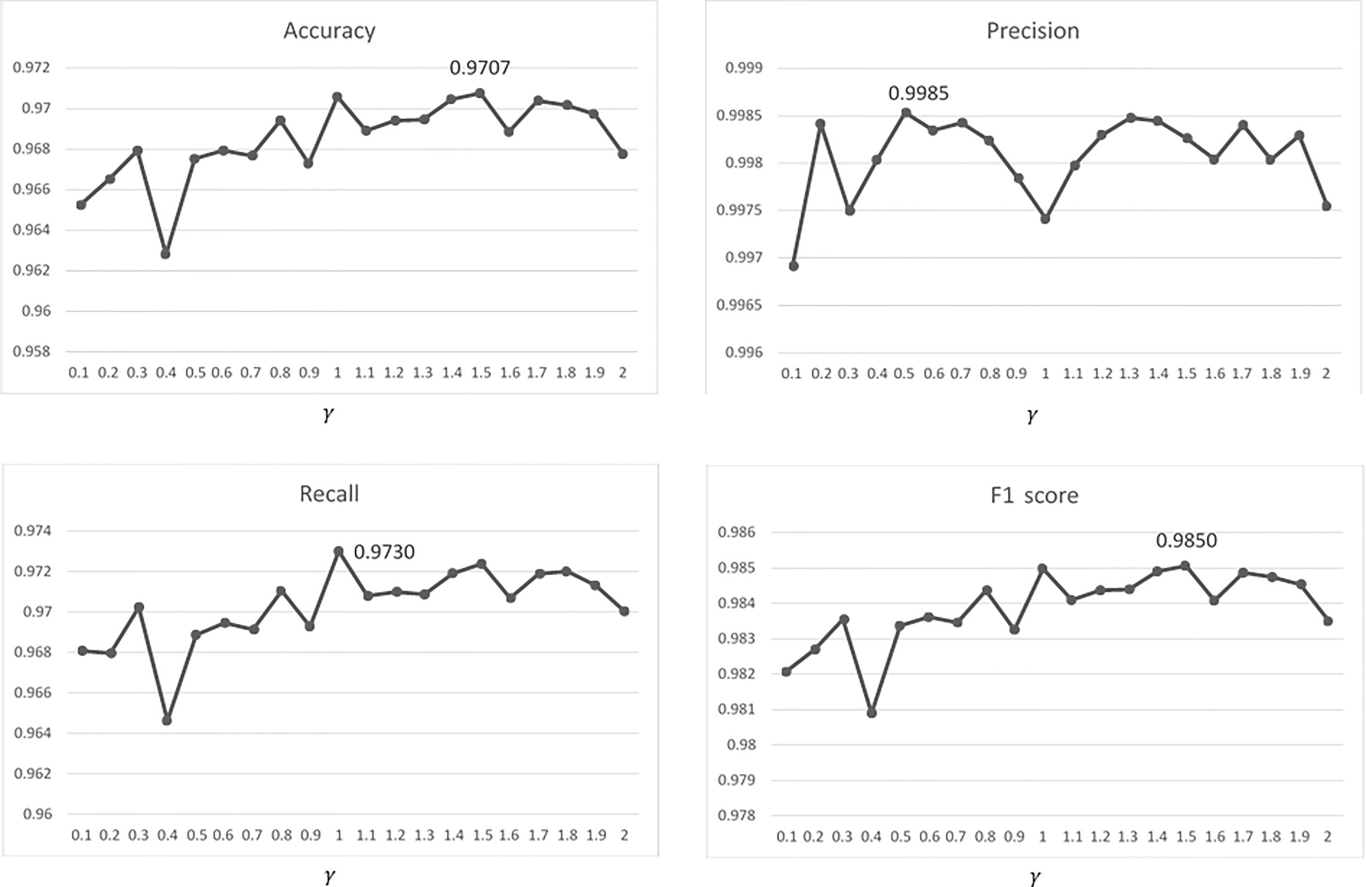
Performances of the leaf segmentation versus different values of gamma (*γ* =0.1~2.0).

**Figure 13:**
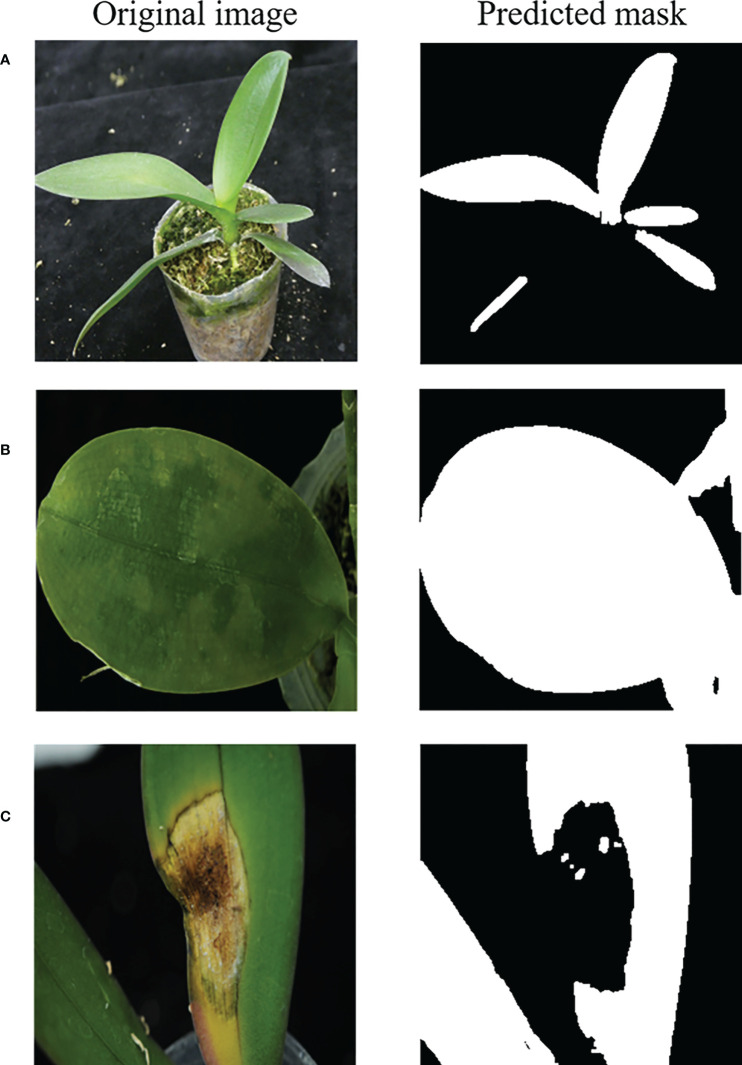
**(A–C)** Predicted masks with the larger errors.

The performances of the leaf segmentation with contrast enhancement (described in section 2.2.1) are shown in **Figure 12** for different gamma *γ* values. The accuracy=0.9707 is highest when *γ* =1.5, and the corresponding precision, recall and F1-score are excellent as listed in the row of (CE + U-net) of **Table 1**. Only three cases gave their predicted masks with the larger errors (accuracy< 0.9) as shown in the right panels of **Figure 13**.

**Table 1:** Performances of the leaf segmentation for *γ* =1.5.

Method	Accuracy	Precision	Recall	F1 score
CE^1^ + U-net^2^	0.9707	0.9982	0.9723	0.9850
CE^1^ + U-net^2^ + RNLFA^3^	0.9707	0.9986	0.9719	0.9850

^1^CE: contrast enhancement.

^2^U-net: leaf region prediction using the U-net model.

^3^RNLFA: removal of non-leaf fragment areas.

The performances of leaf segmentation (using CE, U-net model, and RNLFA) listed in the row of (CE + U-net + RNLFA) in **Table 1**, were also excellent and we adopted this integrated leaf segmentation method to exclude the non-leaf area for favoring the following disease identification.

### 3.2 Performances of the disease identification

#### Feature importance and category distribution

**Figure 14:**
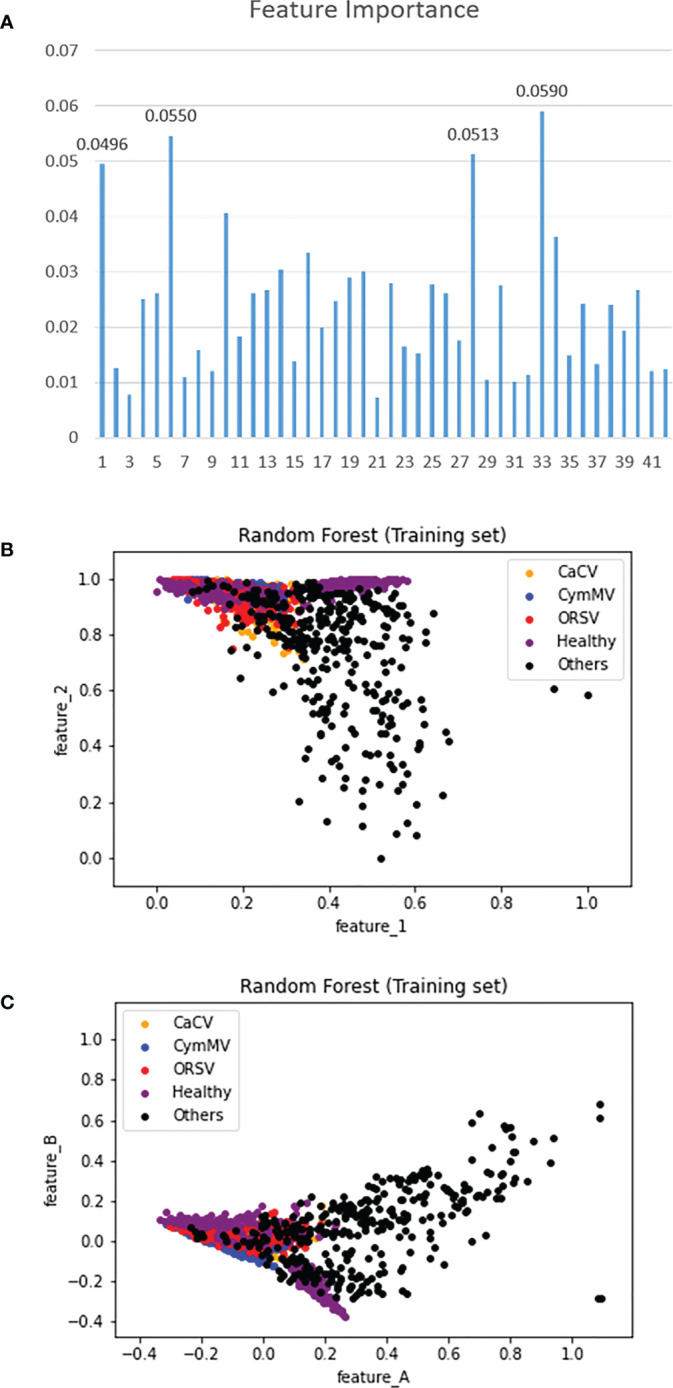
**(A)** The importance of 42 extracted features, **(B)** the category distribution in the top-2 feature plan, and **(C)** the category distribution related to 2 features obtained using the method of PCA with top 4 features.

The relative importance of 42 features was illustrated in (**Figure 14**A). The numbers in the horizontal axis represent the 42 features (contrast, dissimilarity, homogeneity, ASM, energy, correlation, and followed by 36 features of LBP^ri^) extracted using methods of GLCM and LBP^ri^ from the leaf texture. The top four features were contrast and correlation of GLCM as well as the 22^nd^ and 27^th^ of LBP^ri^ features. (**Figure 14B**) presents the distribution of the five categories of orchid leaves associated with the top two features. Furthermore, (**Figure 14C**) shows the category distribution associated with the two features (namely, feature_A and feature_B) after the dimension reduction using PCA (described in section 2.2.2) from the top 4 features shown in (**Figure 14A**). (**Figures 14B, C**) illustrated the distribution of the category “others” differs most from the other four categories. This phenomenon explains the reason why we classified categories into “others” and “non-others” in the first stage of the proposed two-stage model shown in **Figure 10**.

### Performances of the single-stage identification

**Table 2** shows the accuracies of the single-stage identification using the random forest models, designed with different methods of leaf texture feature acquisition (GLCM or/and LBP^ri^) and different gray-level images (obtained from the R, G or B component of each segmented leaf image), to identify the five categories (listed in section 2.1). It was obvious that the best performances were with models designed using GLCM + LBP^ri^ for the leaf texture feature acquisition (the last three rows in **Table 2**). This was the reason for using GLCM + LBP^ri^ to extract leaf texture features in this study. The accuracy (0.87) of the random forest model designed using GLCM + LBP^ri^ with gray-level image of B component (the last row in **Table 2**) was the highest.

**Table 2:** Identification performances of single-stage RF models designed with different combination of texture feature acquisition methods and gray-level images (obtained from the different R, G or B component).

Texture feature acquisition method (gray-level image source)	Accuracy
GLCM (R component)	0.606
GLCM (G component)	0.604
GLCM (B component)	0.677
LBP^ri^ (R component)	0.838
LBP^ri^ (G component)	0.868
LBP^ri^ (B component)	0.859
GLCM+ LBP^ri^ (R component)	0.846
GLCM+ LBP^ri^ (G component)	0.868
GLCM+ LBP^ri^ (B component)	**0.870**

The bold value indicates the highest accuracy.

Identification accuracies of four single-stage models with or without the leaf segmentation are listed in **Table 3**. Furthermore, **Table 4** shows the values of recall and precision by category using single-stage models with leaf segmentation in identifying leaves with the CaCV infection, CymMV infection, ORSV infection, health status, and other diseases.

**Table 3:** Identification accuracies of single-stage models (for identifying five categories of leaf diseases) with or without leaf segmentation.

Single-stage model	Accuracy	
	With leaf segmentation	Without leaf segmentation
RF with GLCM+ LBP^ri^ (R component)	0.846	0.827
RF with GLCM+ LBP^ri^ (G component)	0.868	0.827
RF with GLCM+ LBP^ri^ (B component)	0.870	0.833
InceptionV3	**0.894**	0.883

The bold value indicates the highest accuracy.

**Table 4:** The identification recall and precision of single-stage models with leaf segmentation.

Single-stage model	RF+R component		RF+G component		RF+B component		InceptionV3	
	REC	PRE	REC	PRE	REC	PRE	REC	PRE
CaCV	0.924	0.899	0.925	0.925	0.953	0.910	0.943	0.962
CymMV	0.821	0.716	0.863	0.759	0.853	0.757	0.905	0.851
ORSV	0.585	0.706	0.659	0.740	0.610	0.735	0.768	0.797
Health	0.924	0.944	0.924	0.955	0.935	0.966	0.967	0.927
Others	0.943	0.953	0.943	0.953	**0.966**	**0.966**	0.862	0.915

The bold value indicates the highest recall or precision.

### Performances of the two-stage identification

**Table 5** shows the identification performances for leaf diseases using the proposed two-stage architecture shown in **Figures 2**, **10**, and described in section 2.2. Each bold value in **Table 5** indicates that it outperformed other single-stage models shown in **Tables 4**, **3**, except for the category of “others” which performs equally to the RF model with B-component features shown in **Table 4**. This equal performance resulted from the first stage of the two-stage model was using this RF model with B-component features shown in **Table 4** for preliminary categories identification of “others” and “non-others”.

**Table 5:** Identification performances (the recall, precision and mean accuracy) of the proposed two-stage model.

RF+InceptionV3 (two-stage model)		
	REC	PRE
CaCV	**0.972**	**0.981**
CymMV	**0.905**	**0.851**
ORSV	**0.768**	**0.797**
Health	0.957	**0.978**
Others	**0.966**	**0.966**
Mean accuracy = **0.918**		

Each bold value indicates that the two-stage model outperforms other models (or performs equally to the best model) shown in **Tables 3**, **4**.

**Figure 15:**
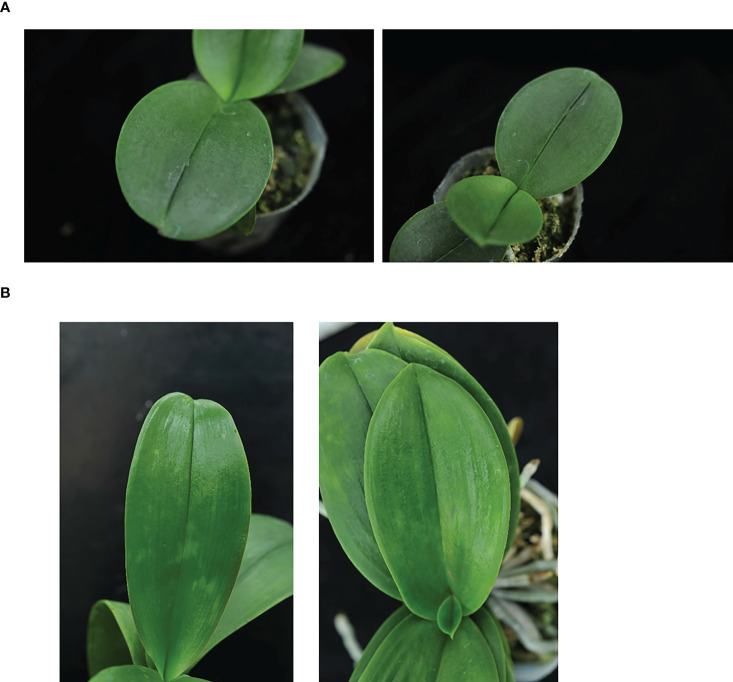
Misidentified examples between two categories, cymbidium mosaic virus (CymMV) and odontoglossum ringspot virus (ORSV). **(A)** ORSV cases misidentified as CymMV. **(B)** CymMV cases misidentified as ORSV.

Based on the experimental results (**Table 5**), it was obvious that the identification performances of two categories (CymMV and ORSV) were the lowest among the five categories. This mainly resulted from the differences between symptoms of the two categories (CymMV and ORSV) were not noticeable or in some cases they were even similar. However, these two categories were confirmed by ELISA assay. Examples of misclassified CymMV and ORSV cases are shown in **Figure 15**. The accuracy comparison in the identification between these two categories (CymMV and ORSV) using the proposed two-stage model and by the expert was performed. The results presented in **Table 6** showed that the proposed two-stage model outperformed expert identification in the identification of the easily misidentified categories (CymMV and ORSV).

**Table 6:** The accuracy comparison between the proposed two-stage model and expert identifications for the CymMV and ORSV categories.

Identification method	CymMV	ORSV	Average
Expert	0.921	0.372	0.667
Two-stage model	0.905	0.768	**0.842**

The bold value indicates the highest average accuracy.

## 4 Discussions

### 4.1 Image preprocessing

The contrast enhancement, including Equation (3), the Y-component of the color space YUV with the color space transformations, and Equations (1)-(2), was adopted for brightness adjustment to solve the non-uniform brightness issue in this study. The effectiveness of brightness adjustment was shown in (**Figures 4B, D**) with different values of gamma *γ* . Moreover, the accuracy, precision, recall, and F1 score of the leaf segmentation (illustrated in **Figure 12**) with different values (0.1-2) of gamma *γ* were all excellent (>0.962). The results described above directly or indirectly proved that the adopted contrast enhancement method solved the issue of non-uniform brightness effectively. Besides the YUV space, other color spaces (such as YC_b_C_r_, HSI, Lab, HSL, etc.) which include one component of brightness, intensity, or lightness might be other options with the corresponding color space transformations for implementing the contrast enhancement to solve the non-uniform brightness issue (Banerjee et al., 2016; Sabzi et al., 2017; Shi et al., 2019; Tan and Isa, 2019; Xiong and Shang, 2020).

### 4.2 Leaf segmentation and single-stage orchid-disease identification

Predicted masks with the larger errors (only three cases were obtained with the accuracy< 0.9 in this study) obtained in the leaf segmentation process as shown in the three right panels of (**Figures 13**A–C) resulted from (a) one of the leaves was confused with the green objects inside the pot, (b) the pot color was close to the colors of leaves, and (c) the leaf was seriously injured (there were only 2 images of this kind of the seriously injured leaf in the entire dataset of this study), respectively.

From our experimental results of the single-stage identification, the identification performance listed in **Table 3** shows that the accuracy of every model with the leaf segmentation (accuracy=0.846-0.894) outperformed the corresponding model without leaf segmentation (accuracy=0.827-0.883). And The inceptionV3 model performed best with accuracy=0.894. The results verified the importance of using the leaf segmentation for accurate identification.

**Table 4** shows that all three RF models with gray-level images of R, G or B component outperform the inceptionV3 model in the first-stage identification results (recall and precision) for the identification of the category “others”. The RF model with 42 texture features extracted from the gray-level image of B component performed best in the identification of the category “others”. This was the reason why we adopted the RF model with B-component texture features as the first stage model in designing the proposed two-stage model (**Figure 10**) to precisely classify two categories of “others” and “non-others”.

### 4.3 Two-stage orchid-disease identification performances and future works

From the identification performances of categories of “non-others” listed in **Table 5**, it is clear that the proposed two-stage model outperformed all single-stage models listed in **Table 4** and **Table 3** (except that the recall=0.957 of health category listed in **Table 5** was slightly lower than that of the single-stage inceptionV3 model with recall=0.967 listed in **Table 4**). For the identification of the category “others”, the proposed two-stage model performed excellently and equally to that of the RF model with B-component features which was adopted as the first stage model in the proposed two-stage model (**Figure 10**) in this study as mentioned above.

In **Table 5**, the overall accuracy (0.918) for the identification of five categories of *Phalaenopsis* orchid leaf status using the proposed two-stage model (RF + InceptionV3) was excellent and higher than that of the one-stage model (inceptionV3 model) with the best accuracy (0.894) among all one-stage models listed in **Table 3**.

In the identification performance of two-stage model listed in **Table 5**, the recall (0.972) and precision (0.981) in the identification of CaCV-infected leaf symptoms were both the highest among the five categories. While the recall (0.768) and precision (0.797) in the identification of ORSV-infected leaf symptoms were both the lowest among the five categories. Based on the confusion matrix of identification results of the proposed two-stage model, it was clear that the most misclassification cases happened between two categories, CymMV and ORSV infections. Symptoms caused by viral infection may vary in *Phalaenopsis* and often depend on the plant genotype, environment, planting, and virus species and isolates. CymMV and ORSV infections are often misidentified by the naked eye due to their relatively unnoticeable and similar symptoms in early stage of infection or different *Phalaenopsis* cultivars (Wong et al., 1996; Seoh et al., 1998; Koh et al., 2014). **Figure 15** shows four examples of misclassification cases (identified using the two-stage model) between CymMV-infected and ORSV-infected categories. CymMV- and ORSV-infected leaves often exhibit mild symptoms in the early stage of infection or in some *Phalaenopsis* cultivars as shown in **Figure 15**.

**Table 6** shows the proposed two-stage model identification (accuracy=0.842) outperformed the expert identification (accuracy=0.667) in the identification of CymMV and ORSV categories (easily misidentified). The excellent identification performances shown in **Tables 5**, **6** demonstrated that the proposed identification system (with architecture shown in **Figure 2**) would be beneficial toward an automatic and accurate disease identification using *Phalaenopsis* orchid leaf image, which in turn would benefit the orchid cultivation industry.

Future study may focus only on the development of more precise identification of the only categories of CymMV- and ORSV-infected leaves by designing a modified model with the third stage. On the other hand, as to identify more infection classes individually and precisely maybe worth trying.

#### Real practice considerations

Plants were placed on a black cloth for photo-taking to verify the feasibility of this developed recognition system. The following options may be considered for building a working system for the real practice in the greenhouse. The first option: an automatic conveyor system (usually with the black plane of a conveyor belt) may be built to take the orchid image for simultaneous disease detection and other cultivation activities by sequentially putting the orchid seedlings or plants on the conveyor belt for the real practice. This should be an easy realization way but may not be smart nor be fully automatic way. The second option: each orchid seedling or plant may be cultivated with a pot surrounded by the black supporting mechanism. The developed disease detection method may be integrated into an APP of a smart device with auto focus function for taking photo of an orchid. Sequentially taking photos of all (or sampled) orchids in a greenhouse by a person or robot (or a moveable robotic arm system) may be considered. The third option: a robot (or a moveable robotic arm system) with automatic orchid detection function can be adopted to sequentially screen all (or sampled) orchid seedlings or plants in a greenhouse. The black cloth or some object like that can be automatically and sequentially placed surrounding each target orchid pot by the robot. Then, the robot can automatically move the camera to the suitable position to take the photo of an orchid seedling or plant for disease detection. The fourth option: similarly, a robot (or a moveable robotic arm system) with automatic orchid detection function can be adopted to sequentially screen all orchid seedlings or plants in a greenhouse. Then, the robot can automatically move the camera to the suitable position to take the photo of an orchid seedling or plant. The non-orchid area (the area without orchid leaf nor its pot in an orchid image) can be identified and removed using an AI model with image processing techniques before performing the disease identification. Any of the above four options for the real practice of the disease identification system may be considered to simultaneously integrate with the original routine cultivation activities, the disease identification method proposed in this study, and a suitable APP in the smart device.

### 4.4 Comparison with associated studies

As mentioned in section 1.1, many previous studies focused on AI models or image processing techniques for the identification of plant diseases. For example, the review paper (Vishnoi et al., 2021) mentioned that the top 3 studied plants were rice (11%), tomato (11%) and corn (7%) in addition to many other plants using image processing or AI methods during 2009-2020. However, orchid disease identification studies using AI or image processing methods shared only 1% (Vishnoi et al., 2021). Based on reports of other review articles (Dhaka et al., 2021; Ghorai et al., 2021) and the survey results, there were very few orchid disease identification studies involved the use of the AI model and leaf images.

Huang (2007) adopted an AI model (back-propagation neural network, BPNN, with lesion area segmentation and texture features) in detecting and classifying *Phalaenopsis* seedling diseases including 4 categories: the bacterial soft rot (BSR), bacterial brown spot (BBS), Phytophthora black rot (PBR), and OK with the average accuracy reached 0.896 (Huang, 2007). The classified diseases (BSR, BBS and PBR) (Huang, 2007) were different from diseases (CaCV infection, CymMV infection, ORSV infection, and others) identified in this study. Furthermore, the image symptoms or texture features of CymMV- and ORSV-infected leaves, shown in (**Figures 1B, C**), are often less obvious than that presented on the leaves of BSR, BBS and PBR diseases (Huang, 2007). The symptoms or texture features of a CymMV- or ORSV-infected leaf, (**Figures 1B, C**), are often not easily identified by the naked eye, even though they were confirmed by ELISA assay. The mean identification accuracy (=0.918) of the current study outperforms that (=0.896) of the previous study (Huang, 2007). Moreover, the recall and precision were not provided, nor the disease type of others was included in previous study (Huang, 2007).

The main difference and the importance of the proposed algorithm versus other state-of-the-art algorithms include the following points. As described above and in section 1.1, many AI models with image processing methods were proposed in detection diseases for different plants in the previous studies (Dhaka et al., 2021; Ghorai et al., 2021; Vishnoi et al., 2021) and reached fair, good, or excellent accuracies (0.590-0.9975) as mentioned in 32 articles (Dhaka et al., 2021). However, around 99% of these previous studies were not associated with the orchid disease (Vishnoi et al., 2021). Furthermore, the image and detection conditions adopted among these previous studies differed a lot. Nevertheless, the mean accuracy (0.918) of the current study reached the excellent detection performance for orchid diseases when compared to that (0.896) of previous studies.

An aforementioned study, the AI model used for orchid disease detection was adopted from a traditional AI model, BPNN, which was used for the detection of two bacterial and one Phytophthora infection symptoms that were obviously identifiable even with the necked eye (Huang, 2007). Moreover, many of their images were taken after fixing the leaves on a plane using pushpins, that could limit the usage for real practice (Huang, 2007). Contrarily, our study designed the more advanced AI model for detecting more common virus diseases of orchid seedlings with symptoms that were less noticeable and often could not be easily identified by the naked eye.

Besides that, the proposed model system of the current study reached an accuracy of 0.842 and outperformed the human expert with an accuracy of 0.667 in the identification of easily misidentified categories between CymMV and ORSV which often showed no noticeable symptoms in the early stage of infection. Therefore, our proposed method should be beneficial to accurately detect the common virus diseases of orchid seedlings for early detection followed by early preventive measures to avoid the extended infection and loss.

## 5 Conclusions

*Phalaenopsis* orchid cultivation is often hindered by viral diseases. The proposed system architecture for the identification of *Phalaenopsis* orchid viral diseases were successfully implemented and reached excellent identification performances. This study conducted a system included designing the U-net model for leaf segmentation with contrast enhancement techniques followed by the approach of removal of non-leaf fragment areas, as well as creating a two-stage model (a random forest model and an InveptionV3 model) with methods of image augmentation and leaf texture feature acquisition from segmented leaf images. This system reached identification accuracy of 0.918 in the identification of five categories of orchid leaves. Moreover, this system provided an accuracy of 0.842 and outperformed the human expert with an accuracy of 0.667 in the identification of easily misidentified categories between CymMV and ORSV infections. In the future, we will continue to provide more database images to improve the recognition accuracy of CymMV and ORSV categories. We believe this outcome would serve as a solid ground for the development of the accurate, automatic, and cost-effective disease identification system for supporting orchid cultivation industry.

## Data availability statement

The original contributions presented in the study are included in the article/supplementary material. Further inquiries can be directed to the corresponding authors.

## Author contributions

F-JJ, and Y-KC conceived the study. Y-KC, and F-JJ designed the approach and performed the computational analysis with C-FT. C-HL, S-SY, Y-KC and F-JJ supervised the work and tested the program. F-HW, C-FT, Y-KC, C-WL, and F-JJ wrote the manuscript. C-HL, C-HH, C-FT, and F-JJ collected the data. C-FT, C-HL, C-HH, F-HW, C-HL, S-SY, Y-KC, and F-JJ contributed analyzing experimental studies. All authors read and approved the final manuscript. C-FT, C-HH, and F-HW contributed equally and are the first authors. Y-KC, and F-JJ contributed equally and are the correspondents. All authors contributed to the article and approved the submitted version.

## Funding

This research was funded by the Advanced Plant Biotechnology Center from The Featured Areas Research Center Program within the framework of the Higher Education Sprout Project by the Ministry of Education (MOE) in Taiwan.

## Acknowledgments

We are grateful to Dr Chung-Jan Chang, Professor Emeritus of the University of Georgia, for his critical review of the manuscript.

## Conflict of interest

The authors declare that the research was conducted in the absence of any commercial or financial relationships that could be construed as a potential conflict of interest.

## Publisher’s note

All claims expressed in this article are solely those of the authors and do not necessarily represent those of their affiliated organizations, or those of the publisher, the editors and the reviewers. Any product that may be evaluated in this article, or claim that may be made by its manufacturer, is not guaranteed or endorsed by the publisher.
